# Safety of COVID-19 mRNA Vaccines in Patients with Cancer Enrolled in Early-Phase Clinical Trials

**DOI:** 10.3390/cancers13225829

**Published:** 2021-11-20

**Authors:** Pamela Trillo Aliaga, Dario Trapani, José Luis Sandoval, Edoardo Crimini, Gabriele Antonarelli, Grazia Vivanet, Stefania Morganti, Chiara Corti, Paolo Tarantino, Alex Friedlaender, Carmen Belli, Ida Minchella, Marzia Locatelli, Angela Esposito, Carmen Criscitiello, Giuseppe Curigliano

**Affiliations:** 1 Division of New Drugs and Early Drug Development, European Institute of Oncology IRCCS, 20141 Milan, Italy; Pamela.TrilloAliaga@ieo.it (P.T.A.); dario.trapani@ieo.it (D.T.); edoardo.crimini@ieo.it (E.C.); Gabriele.Antonarelli@ieo.it (G.A.); grazia.vivanet@ieo.it (G.V.); stefania.morganti@ieo.it (S.M.); Chiara.Corti@ieo.it (C.C.); Paolo.tarantino@ieo.it (P.T.); carmen.belli@ieo.it (C.B.); ida.minchella@ieo.it (I.M.); marzia.locatelli@ieo.it (M.L.); angela.esposito@ieo.it (A.E.); carmen.criscitiello@ieo.it (C.C.); 2 Department of Oncology and Hematology, University of Milan, 20122 Milan, Italy; 3 Unit of Population Epidemiology, Division and Department of Primary Care Medicine, Geneva University Hospitals, 1205 Geneva, Switzerland; jose.sandoval@cantab.net; 4 Department of Oncology, Geneva University Hospitals, 1205 Geneva, Switzerland; alex.friedlaender@hcuge.ch

**Keywords:** SARS-CoV-2, COVID-19, COVID and cancer, early-phase clinical trials, novel immunotherapy, COVID-19 vaccine, phase one trial, targeted therapy, solid tumors

## Abstract

**Simple Summary:**

We investigated for the first time the safety profile of COVID-19 vaccines in patients receiving new antineoplastic agents in early-stage clinical trials, including new immuno-regulatory anti-cancer investigational compounds and drug combinations. We found that about three-quarters of the patients under active anticancer treatments experienced mild to moderate adverse effects (AEs) related to COVID-19 vaccines. Patients enrolled in early-phase trials or receiving experimental immunotherapy agents did not experience worse AEs related to the vaccine than patients with cancer not enrolled in these trials, receiving approved drugs. The safety profile of COVID-19 vaccines in patients enrolled in early-phase clinical trials, including those treated with new immune checkpoint inhibitors, does not seem to differ from that of the general population of patients with cancer. Our data support the current vaccine prioritization of all cancer patients with active treatment and calls for data sharing from vaccinated patients enrolled in early-phase clinical trials.

**Abstract:**

Pivotal trials of COVID-19 vaccines did not include cancer patients, with questions remaining about their safety and efficacy in this population. Patients enrolled in early-phase clinical trials receive novel treatments with unknown efficacy and safety profiles. Studies on the safety of COVID-19 vaccines in these patients are urgently required. This is a retrospective, real-world, cohort study of patients receiving anticancer treatments and COVID-19 vaccines between 1 February and 25 June 2021 at the Division of New Drugs Development for Innovative Therapies of the European Institute of Oncology. One hundred thirteen patients were enrolled, 40 in early-phase clinical trials, and 20 under novel immunotherapy agents. Nearly three-quarters of the patients experienced at least one adverse event (AE) after the first dose (1D) (74.3%) and second dose (2D) (72.6%). Most of the AEs were local (67.3% 1D and 61.9% after 2D), while 31.8% (1D) and 38.1% (2D) of the patients had systemic AEs. No AEs above grade 2 were observed. Therefore, COVID-19 vaccines appear to be safe in patients enrolled in early-phase clinical trials, including patients receiving novel immunotherapy compounds. All cancer patients should be prioritized for COVID-19 vaccination, regardless of ongoing treatments or enrollment in early-phase trials.

## 1. Introduction

The rapid development of vaccines against severe acute respiratory syndrome coronavirus-2 (SARS-CoV-2) has represented an unprecedented effort in the fight against a pandemic with profound global health and socioeconomic repercussions [1]. The pivotal trials of coronavirus disease 2019 (COVID-19) vaccines have shown efficacy in 70–90% of individuals, with an overall favorable safety profile in healthy individuals, the elderly, and those with chronic diseases [2,3,4,5,6]. Certain patients with cancer are vulnerable and at increased risk of severe COVID-19 outcomes [7,8,9]. Therefore, they were identified as a priority population for vaccination, despite their initial exclusion from the pivotal vaccine clinical trials [10]. Presently, different types of COVID-19 vaccines have been authorized for human use in Europe and the USA. Two are mRNA-based (i.e., Pfizer-BioNTech (Cambridge, MA, USA) [2] and Moderna (Cambridge, MA, USA) [3,4,5]), and two are viral vector-based (i.e., Oxford–AstraZeneca (Wilmington, DE, USA) [6] and Johnson & Johnson (New Brunswick, NJ, USA) [11]). While their capacity to induce an effective immune response is well-documented in clinical trials, the immunogenicity and reactogenicity in patients with tumors, particularly in those treated with immuno-regulating drugs, is still the object of intense research. Data reported in this regard stem from non-randomized trials and small observational studies [12]. For example, patients with hematological or auto-immune diseases treated with anti-CD20 agents or undergoing stem cell transplantation seem to have lower levels of seroconversion than the overall population, due severe immunosuppression [13]. On the contrary, most patients with solid tumors being treated with chemotherapy or immunotherapy achieve seroconversion after a full course of vaccines [14]. Patients with cancer are treated with various immuno-modulating agents, either immune-enhancing or -suppressing. However, the side effect profiles of COVID-19 vaccines when administered concomitantly to new antineoplastic agents, especially with new immunotherapy drugs, are still unknown: for instance, hyperactive immune responses, such as cytokine storms, were reported [15]. Similarly to all other patients with cancer, those enrolled in clinical trials are a priority vaccination group [16]. Pursuing a risk-adapted strategy and in the weight of risk-benefit amidst pandemic, there has been no exclusion of patients enrolled in early-phase clinical trials during the COVID-19 pandemic from the vaccination programs. Early-phase clinical trials test relatively new molecules or drug combinations, most often with incompletely described or unknown pharmacokinetic or pharmacodynamic and safety profiles. Furthermore, patients enrolled in early-phase clinical trials are frequently heavily pretreated and have metastatic disease, meaning they are potentially more vulnerable than other patients with cancer. As such, their prioritization to receive the vaccination is unquestionable [17,18]. 

This work aimed to describe the safety profile of COVID-19 vaccines in patients receiving novel antineoplastic drugs in early-phase clinical trials, compared to other vaccinated cancer patients undergoing standard treatments, with a particular focus on those receiving new immunotherapy agents [18].

## 2. Materials and Methods

### 2.1. Patient Recruitment and Data Collection

We performed a retrospective study, including all vaccinated patients older than 18 years of age with any cancer at any stage undergoing treatment in our department (Division of New Drugs and Early Drug Development, European Institute of Oncology, IRCCS) between 1 February and 25 June 2021. We included patients who had received one or two doses of COVID-19 vaccines among the ones approved and available in Italy at the time (i.e., Pfizer-BioNTech [BNT162b2], Moderna [mRNA-1273], and AstraZeneca [ChAdO×1 nCoV-19]). Experimental COVID19 vaccines, either for type and schedule, were not considered for the purpose of this analysis. Patients were included regardless of whether they were participating in clinical trials or receiving standard treatments. We excluded patients who refused the COVID-19 vaccine or opted to postpone its administration. We also excluded patients referred to our department for follow-up only (i.e., not under treatment).

We collected the following patient demographic, pathological, and clinical characteristics: age, gender, performance status, smoking habits, type of cancer, TNM stage, comorbidities, type of ongoing anticancer treatments, number of previous lines of therapy received, and concomitant steroid use. All patients were administered the COVID-19 vaccine by their oncologist in our institution, according to the local immunization guidelines. After vaccination, the patients were contacted by telephone within one week, to enquire about early adverse events (AEs). The patients were subsequently asked about new AEs during the scheduled follow-up visits for the continuation of cancer treatment. We report both local (e.g., pain, erythema, edema, induration at the injection site, and locoregional reactive lymphadenopathies) and systemic AEs (i.e., fever, chills, headache, fatigue, myalgia, arthralgia, nausea, vomiting, and others), after the first and the second dose of COVID-19 vaccine. AEs were graded according to Common Terminology Criteria for AEs (CTCAE) version 5.0 [19].

### 2.2. Ethics Statement 

The study conforms with the Helsinki Declaration for research with humans and adheres to the good clinical practice guidelines and the Italian legislation (DM 15/07/97 and amendments), including the exceptions and exemptions related to the COVID-19 pandemic (EU/EMA/GCP-IWG/CTFG/CTEG/HMA and AIFA guidelines for clinical research of the 28 April 2020, in EudraLex Volume 10 Clinical trials). A research protocol for retrospective studies was submitted and approved by our IRB (approval number: UID 3031). Data were anonymized and collected with data minimization. Only the investigators are aware of the de-codification encryption to re-identify patients. The data are stored in the institutional dataset of medical records for research, protected with a password, and accessible only from hospital-based computers, provided an identification as a doctor, nurse, or data management personnel of our clinical unit.

### 2.3. Endpoint and Outcome 

The primary endpoint of the study was to describe the safety profile of COVID-19 vaccines in our cohort. The primary outcome was the incidence of COVID-19 vaccine-related AEs, as assigned by the treating physicians and/or reported by the patient. The analyses were performed in the overall study population and in the subgroups of patients enrolled in early-phase clinical trials (any treatment) or receiving experimental immunotherapy drugs. 

### 2.4. Statistical Analysis 

We present absolute and relative frequencies of the participants’ outcomes and clinicodemographic characteristics, overall and in the previously mentioned subgroups. In addition, we examined the association between patient variables and the primary endpoint using a univariate logistic regression model, calculating the odds ratio (OR) as a measure of association. Due to the low numbers of events, a multivariate model could not be implemented. We present logOdds ratios in figures (logOR) due to the ease of the graphical representation. Calculated *p* values were 2-sided, and a *p* < 0.05 was considered statistically significant. Data were analyzed and processed using R (version 4.1.0) and Stata (version 15.0).

## 3. Results

### 3.1. Demographic and Clinical Characteristics of the Overall Population 

We included 113 patients in the final analysis. The main clinical and demographic characteristics are reported in Table 1. Among the 35% of patients enrolled in an early-phase clinical trial (*n* = 40), half was receiving an experimental immuno-oncology agent (*n* = 20). Eighty-two percent of the patients were female (*n* = 96), and the median age was 60 (interquartile range: 53–69). Most patients were never smokers (76.1%, *n* = 86) and had at least one comorbid condition (81.4%, *n* = 92). The majority of patients had breast cancer (76.1%, *n* = 86), followed by lung cancer (6.2%, *n* = 7) and melanoma (5.3%, *n* = 6). The disease distribution was different in the early-phase trial cohort, with only approximately a third of patients with breast cancer (35%, *n* = 14). Close to 98% of the patients (*n* = 110) had metastatic disease. Half received first- and second-line treatments (52.2%, *n* = 59), the other half third-line treatment or beyond (47.8%, *n* = 54). The most common treatments were single-agent targeted therapy (23.9%, *n* = 27), chemotherapy (21%, *n* = 25), and immunotherapy (15.9%, *n* = 18). However, the patients enrolled in phase 1 and 2 clinical trials received mainly targeted agents (43%, *n* = 17) and immunotherapy (45%, *n* = 18).

### 3.2. Type of COVID-19 Vaccine 

The majority of patients received an mRNA-based vaccine: 59 patients received Moderna (52.2%), 52 received Pfizer (46.6%), and 2 received AstraZeneca (1.8%) vaccines. In the cohort of early-phase trials patients, 22 received Pfizer (55%), 18 received Moderna (45%), and none received AstraZeneca vaccine. The same distribution was observed in the subset of patients treated with novel immunotherapies. All patients received the two doses of the vaccines.

### 3.3. Safety 

#### 3.3.1. Safety in the Overall Population 

**Figure 1:**
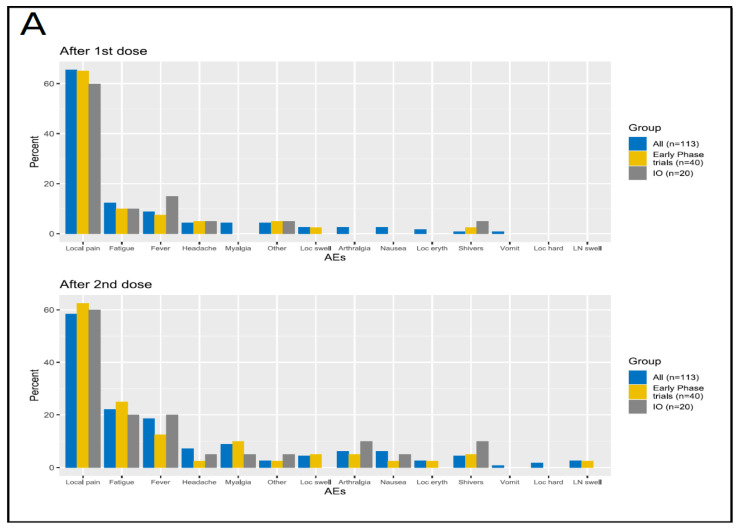

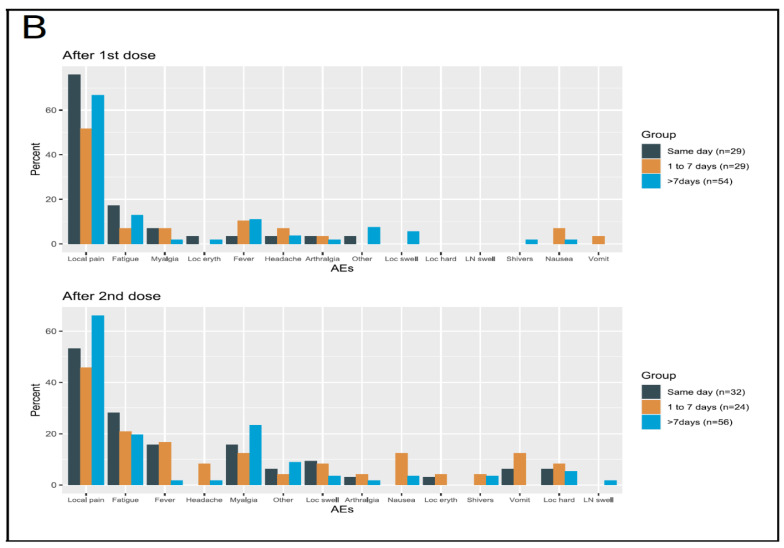
Incidence of AEs after the first and the second dose (**A**) Incidence of adverse events after the first and the second dose in all patients, in those enrolled in early-phase trials and in patients receiving immunotherapy. Immunotherapy cohort (IO), lymph node swelling (LN swell), local swelling (Loc swell), local hardening (Loc hard), local erythema (Loc eryth). (**B**). Incidence of adverse events after the first and the second dose, according to the interval between the vaccine administration and the previous antineoplastic treatment. Lymph node swelling (LN swell), local swelling (Loc swell), local hardening (Loc hard), local erythema (Loc eryth).

**Figure 2:**
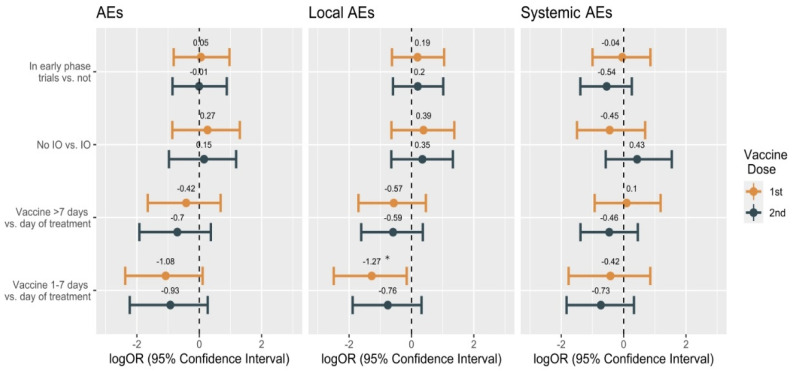
Association of the overall, local and systemic AEs to multiple variables. Association between the incidence of adverse events after each vaccine dose and being enrolled in an early-phase trial, treatment with immunotherapy, and interval between vaccination and previous antineoplastic treatment. Immunotherapy cohort (IO), patients that did not receive immunotherapy (No-IO), log odds ratio (logOR). * *p* = 0.03.

**Figure 3:**
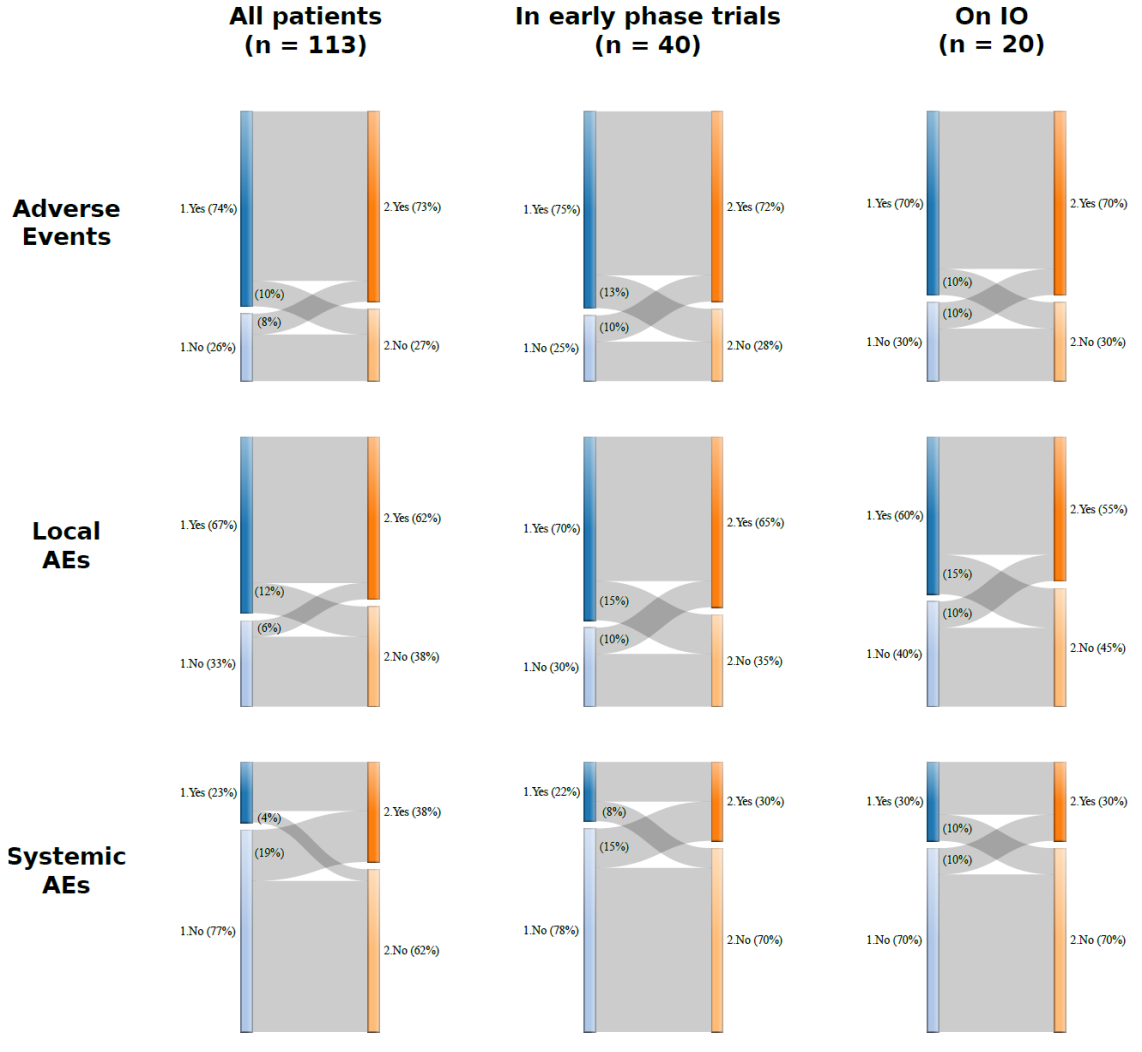
Incidence of the AEs after the first and the second dose of vaccine. The first dose is indicated with the blue bar, the second dose with the orange bar. Changes in the occurrence of adverse effects after the first and the second dose of vaccine, in all patients, in those enrolled in early-phase trials and in patients treated with immunotherapy. Immunotherapy cohort (IO), adverse effects (AEs).

In the overall cohort of patients (*n* = 113), 74.3% (*n* = 84) experienced at least one AE, after the first inoculum. Thereof, 76 patients (67.3%) had local AEs, and 26 (23.0%) had systemic AEs. After the second dose, 82 patients (72.6%) reported at least one AE, of which 70 patients (61.9%) with local AEs, and 43 (31.8%) with systemic AEs (Table 1). All the AEs were grade 1 (mild) and 2 (moderate). No grade 3 or higher AEs were observed. The most common AEs were local pain (65.5%, *n* = 74), fatigue (12.4%, *n* = 14), and fever (8.8%, *n* = 10) after the first dose. Local pain (58.4%, *n* = 66), fatigue (22.1%, *n* = 25), fever (18.6%, *n* = 21), and myalgia (8.8%, *n* = 10) were the most common AEs reported after the second dose (Figure 1A, Supplementary Table S1). We investigated the possible influence of the interval between the vaccine inoculum and the previous cancer treatment (Figure 1B). For the first vaccination dose, 29 patients (25.9%) received the antineoplastic therapy the same day as their vaccination, and 29 (25.9%) during the 7 preceding days. In the remaining patients (48.2, *n* = 54), cancer treatment was administered more than 7 days before vaccination. A similar distribution of intervals between previous cancer treatment and vaccination was observed for the second vaccination dose (Supplementary Table S2). Overall, we did not observe an association between the incidence of AEs and the interval between the vaccination and the previous cancer therapy. The only statistically significant association between this variable and the outcomes was a decreased probability of local AEs after the first vaccination dose in the group of patients with cancer treatment during the 7 days before vaccination versus those vaccinated on the same day as the treatment (OR: 0.3; 95% CI: 0.1–0.9; *p* = 0.03) (Figure 2, Supplementary Table S3). Most patients with systemic AEs also experienced local AEs (Supplementary Figure S1). After the first dose, the main co-occurring AEs were local pain and fatigue (*n* = 11) and/or fever (*n* = 6). Similarly, after the second dose, the main co-occurring AEs were local pain and fatigue (*n* = 18) and/or fever (*n* = 10). We sought to describe the individual differences in AEs incidence between the first and the second vaccine doses using Sankey flow diagrams (Figure 3). We observed that the majority of the patients that had AEs after the first vaccine dose also had AEs after the second dose. We observed similar results for local and systemic AEs. Only 14% of the patients reported no AEs after either vaccine dose, with 27% of the patients reporting no local AEs, and 58% no systemic AEs after either vaccine doses. A similar pattern of AEs was observed for patients enrolled in early-phase clinical trials and for those being treated with novel immunotherapy agents. 

#### 3.3.2. Safety in the Cohort of Patients Enrolled in Early-Phase Clinical Trials

Seventy-five percent (*n* = 30) of the patients treated in early-phase clinical trials experienced at least one AE after the first dose. Of these, 28 patients (70.0%) had local AEs, and 9 (23.0%) reported systemic AEs. After the second dose, 73% of the patients (*n* = 29) experienced at least one AE, with 26 (65.0%) and 12 (30.0%) reporting local and systemic AEs, respectively (Table 1). All the patients had grade 1–2 AEs. After the first dose, the most common AE types were local pain (65.0%, *n* = 26), fatigue (10.0%, *n* = 4), and fever (7.5%, *n* = 3). After the second dose, local pain (62.5%, *n* = 25), fatigue (25.0%, *n* = 10), fever (12.5%, *n* = 5), and myalgia (10.0%, *n* = 4) were the most common AEs, like in the overall population (Figure 1A, Supplementary Table S1). AE co-occurrence patterns and temporal flows were similar to those of the overall population (Supplementary Figure S1, Figure 3). 

#### 3.3.3. Safety in the Group Receiving Experimental Immunotherapy Drugs

Seventy percent of the patients (*n* = 14) treated with novel immunotherapy drugs experienced at least one AE after the first dose. Twelve patients (60.0%) had local AEs, and 6 (30.0%) reported systemic AEs. Similarly, after the second dose, 70% of the patients (*n* = 14) experienced at least one AE, with 11 (55.0%) reporting local AEs, and 6 (30.0%) systemic AEs (Table 1). The most common AEs were local pain (65.0%), asthenia (10.0%), and fever (7.5%) after the first dose, and local pain (62.5%), asthenia (25.0%), and fever (12.5%) after the second dose (Figure 1A, Supplementary Table S1). As with participants enrolled in early-phase clinical trials, participants undergoing treatment with novel immunotherapy agents displayed similar AEs co-occurrence patterns and temporal flows to those of the overall population (Supplementary Figure S1, Figure 3). As hinted by the descriptive results, no association was observed between the participation in early-phase clinical trials or the treatment with novel immune checkpoint inhibitors and the occurrence of AEs (overall, local, or systemic) after the first (ORAEs = 1.1 [0.4;2.6] *p* = 0.9, ORlocal AEs = 1.2 [0.5;2.9] *p* = 0.65, ORsystemic AEs = 1 [0.4;2.4] *p* = 0.92) or the second dose (ORAEs = 1 [0.4;2.4] *p* = 1, ORlocal AEs = 1.2 [0.6;2.8] *p* = 0.62, ORsystemic AEs = 0.6 [0.2;1.3] *p* = 0.19) (Figure 2).

## 4. Discussion

Due to their vulnerability, patients with cancer are a priority group for COVID-19 vaccination [12]. However, the interaction between different COVID-19 vaccine types and antineoplastic treatments is largely unknown. The overall safety and activity profile of COVID-19 vaccines is not well characterized, since patients with cancer were either systematically excluded or have a low representation in the pivotal vaccine efficacy trials [20]. This paradox regarding the low accrual of patients with cancer in vaccination trials and their prioritization for vaccination strengthens the need to collect, analyze, and share real-world data [21]. Real-world-data-based studies could prompt actions if new vaccine efficacy and safety signals are identified in specific population subgroups, such as patients with cancer and those included in early-stage clinical trials. Studies of seroconversion after COVID-19 vaccines have portrayed a spectrum of diverse immune responses, which seems to be affected only in part by the treatment type and by disease type and stage, sex, and age. While vaccine immunogenicity in patients with cancer may be, for the most part, similar to that of the general population, very limited evidence is available on vaccines’ safety when used concomitantly with antineoplastic treatments, and no data have been collected regarding vaccines’ safety for patients administered experimental immuno-modulating compounds. The consensus guidelines of the main international oncology societies and relevant stakeholders unequivocally prioritize COVID-19 vaccination in patients enrolled in clinical trials, including early-phase trials. Early drug development is a setting of uncertainty and *in fieri* knowledge discovery, with the possibility of administering concomitant treatments for other diseases (e.g., a vaccine) being selective, very limited, or even non-existent. In the context of the pandemic, all the principal study protocols have been amended to allow the COVID-19 vaccination, based on the principle of beneficence, despite the absence of clear evidence of vaccine safety in this setting [22]. There has been a consensus that the best vaccine is the one available at a specific time and place and that earlier vaccination is better than later, with few exceptions derived from pharmacovigilance and restrictions related to non-cancer diseases [23]. Our study reports the first real-world analysis of a group of patients enrolled in early-phase clinical trials as part of a larger cohort of patients with cancer. We analyzed the safety profiles in the overall cohort and in subsets of patients receiving experimental antineoplastic drugs, including novel immunotherapy agents. We reported no relevant difference in the safety profiles across the subgroups. The most common grade 1 and 2 reactions were related to the local inoculation, with transient systemic AEs. Our study supports the body of evidence suggesting the safety of COVID-19 vaccines in patients receiving various cancer treatments and supports the vaccination prioritization of patients with cancer in any setting of cancer care [17]. In particular, the vaccines’ safety profile during immunotherapy with novel agents does not seem to differ from that observed with the use of approved antagonists of Programmed Cell Death Protein 1 (PD-1) and its ligand PD-L1 [24]. A plethora of novel immune-enhancing immunotherapy agents that aim to potentiate the T-cell immune-response is being tested in early-phase trials [25], posing a theoretical risk of cytokine release syndrome and severe immuno-mediated organ dysfunctions [26,27]. These events may be too rare to be captured in our analysis and would require longitudinal pharmacovigilance ad hoc studies to be comprehensively collected [28]. However, our study can reassure patients with cancer and healthcare professionals of the safety of COVID-19 vaccination during concomitant cancer treatment. Another key finding of our study relates to the interval between the inoculum and previous treatment administration. Univariate logistic regression models did not identify an association between this interval and the occurrence of AEs, except for a potentially decreased probability of local AEs if the vaccine is administrated within 7 days of the last treatment rather than on the same day of the cancer treatment. However, due to the low number of events, the models used are not multivariate. Thus, this association could be spurious or explained by confounding variables and warrants further investigation. Understanding the influence of cancer treatments on the immunization trajectory is now an urgent need, as a subset of patients receiving chemotherapy or immunotherapy may be less likely to seroconvert than the overall population [29]. This can be particularly true among those who received the vaccine within 15 days of cancer treatment, including immune checkpoint inhibitors. The study of the relationship between immune response, immunoglobulin titers, and AEs is the object of several longitudinal studies, but few are the studies conducted specifically in patients enrolled in early-phase clinical trials. Therefore, pooling real-world data regarding the safety and activity of the different COVID-19 vaccines in cancer patients must occur cogently, to identify areas of unmet needs and dissect the inherent contextual complexities. 

Our study presents several limitations. First of all, our population was heterogeneous, and the sample size was small:confounding factors such as cancer type, cancer treatment, and vaccine type could not be accounted reliably in our statistics. Furthermore, the small population and low event numbers hampered the use of multivariate models to better describe the associations between explanatory and outcome variables. For instance, it was not possible to determine the impact of steroid use (either as a treatment or as a premedication for intravenous chemotherapy). However, except for one of the tested associations, all were non-significant at the univariate analysis. In addition, the short follow-up interval and small cohort limited the potential identification of rare events, for example, the occurrence of exceptional toxicities of special interest, and their delayed occurrence. AEs were determined by patients’ self-report one week after vaccination and at the next medical follow-up appointment; therefore, recall bias was possible, although the interval window was not very large. The follow-up time was narrow and did not allow the detection of later toxicities (subacute and chronic AEs) on the longer term or the identification of effects on treatment efficacy. This is a general issue with vaccine development. All patients in our series received mRNA vaccines, with possible problems of generalizability for viral-vector or other types of vaccines. The choice of mRNA vaccines for patients with cancer was determined by the national policy-makers, recommending this type for the most vulnerable populations. Apart from one patient with a dendritic cell neoplasm, no other patients were included with hematological malignancies. Finally, the monocentric nature of the study may hinder its external validity. The strength of this study is that, for the first time, the safety profile of COVID-19 vaccines was investigated in patients receiving new antineoplastic agents in early-stage clinical trials, particularly, in patients receiving new investigational immunoregulatory anticancer compounds and drug combinations. Another strong point is the analysis carried out to assess whether there was a correlation between AEs and the time between administration of the vaccine and administration of the cancer therapy, almost never specified in works of this kind.

## 5. Conclusions

The safety profile of COVID-19 vaccines in patients enrolled in early-phase clinical trials, including for new immune checkpoint inhibitors, does not seem to differ from that of the general population of patients with cancer. Considering the high morbidity and mortality associated with COVID-19 in patients with cancer receiving active treatments, our data support the current vaccine prioritization of all cancer patients with active treatment and calls for data sharing from sponsored early-phase clinical trials to improve the knowledge on vaccine safety and efficacy in this particular subgroup of patients.

## Figures and Tables

**Table 1:** Principal characteristics of the study population. Abbreviations: IQR: inter-quartile range. IO: immunotherapy. ECOG: Eastern Cooperative Oncology Group. AE, AEs: adverse events. Pts: patients. (a) Nine patients had COVID-19 in the year 2020, and only one had the disease within 3 months from the first dose of the vaccine, (b) gastrointestinal (*n* = 6); genitourinary (*n* = 4); thyroid (*n* = 1); cancer of unknown primary (*n* = 1); mesothelioma (*n* = 1); dendritic cell neoplasm (*n* = 1), (c) 29 pts received inhibitors of the Cyclin-Dependent Kinase 4 and 6 and (CDK4/6i), and 2 pts received everolimus + exemestane, (d) included PARPi (*n* = 6); anti-RET (*n* = 6); anti-ALK (*n* = 1); anti-EGFR+anti-PIK3 (*n* = 1); antibody–drug-conjugate anti-HER2 (*n* = 1); anti-CD123 (*n* = 1); selective estrogen receptor degrader (SERD) + CDK4/6i (*n* = 1), (e) included a new generation of anti-PD1 (*n* = 8); anti-ICOS+anti-PDL1 (*n* = 4); anti-PD1+ anti-TIGIT(*n* = 3); anti-PD1+anti-CTL4+anti-TIGIT (*n* = 1); anti-PD1+anti-LAG3 (*n* = 1); anti-PD1+anti-LAG3+A2AR antagonist (*n* = 1); anti-PDL1+anti-FGFR (*n* = 1); IL12-L19L19 (*n* = 1).

	All Patients	In Early-Phase Clinical Trials	Experimental IO ^e^
**N**	113 (100%)	40 (35.4%)	20 (17.7%)
**Sex**			
Male	17 (15.0%)	16 (40%)	11 (55%)
Female	96 (85.0%)	24 (60%)	9 (45%)
**Age, median (IQR)**	60 (53, 69)	59 (49, 67)	58 (50, 66.5)
**Smoking status**			
Never smoker	86 (76.1%)	29 (73%)	12 (60%)
Smoker	19 (16.8%)	8 (20%)	5 (25%)
Former smoker	8 (7.1%)	3 (8%)	3 (15%)
**History of previous COVID-19**			
Yes ^a^	10 (8.8%)	7 (17.5%)	3 (15%)
No	103 (91.2%)	33 (82.5%)	17 (85%)
**Comorbidities**			
No	21 (18.6%)	8 (20%)	4 (20%)
Yes	92 (81.4%)	32 (80%)	16 (80%)
**Obesity**			
No	102 (90.3%)	37 (93%)	18 (90%)
Yes	11 (9.7%)	3 (8%)	2 (10%)
**Tumor type**			
Breast	86 (76.1%)	14 (35%)	5 (25%)
Lung	7 (6.2%)	7 (18%)	1 (5%)
Melanoma	6 (5.3%)	6 (15%)	6 (30%)
Other ^b^	14 (12.4%)	13 (33%)	8 (40%)
**TNM Stage**			
I-III	3 (2.6%)	0 (0%)	0 (0%)
IV	110 (97.4%)	40 (100%)	20 (100%)
**Number of previous lines of treatment**			
0	29 (25.7%)	11 (28%)	5 (25%)
1	30 (26.5%)	13 (33%)	9 (45%)
2	19 (16.8%)	6 (15%)	2 (10%)
3	13 (11.5%)	2 (5%)	1 (5%)
>3	22 (19.5%)	8 (20%)	3 (15%)
**Ongoing treatment**			
Chemotherapy	25 (22.1%)	2 (5%)	0 (0%)
Chemotherapy + Targeted therapy	2 (1.8%)	1 (3%)	0 (0%)
Endocrine Treatment alone	8 (7.1%)	0 (0%)	0 (0%)
Endocrine Treatment + Targeted Therapy ^c^	31 (27.4%)	0 (0%)	0 (0%)
IO	18 (15.9%)	18 (45%)	18 (90%)
IO + Targeted Therapy	2 (1.8%)	2 (5%)	2 (10%)
Targeted Therapy	27 (23.9%)	17 (43%) ^d^	0 (0%)
**Performance Status (ECOG)**			
0	94 (83.2%)	32 (80%)	16 (80%)
1	17 (15.0%)	8 (20%)	4 (20%)
2	2 (1.8%)	0 (0%)	0 (0%)
**Steroids use**			
No	106 (93.8%)	37 (92.5%)	18 (90%)
Yes	7 (6.2%)	3 (7.5%)	2 (10%)
**Type of Vaccine**			
AstraZeneca	2 (1.8%)	0 (0%)	0 (0%)
Moderna	59 (52.2%)	18 (45%)	8 (40%)
Pfizer	52 (46.0%)	22 (55%)	12 (60%)
**After first dose of vaccine**			
AEs	84 (74.3%)	30 (75%)	14 (70%)
Local AEs	76 (67.3%)	28 (70%)	12 (60%)
Systemic AEs	26 (23.0%)	9 (23%)	6 (30%)
**After second dose of vaccine**			
AEs	82 (72.6%)	29 (73%)	14 (70%)
Local AEs	70 (61.9%)	26 (65%)	11 (55%)
Systemic AEs	43 (38.1%)	12 (30%)	6 (30%)

## Data Availability

The datasets analyzed for the purpose of this data analysis are not publicly available. However, data can be obtained from the corresponding author upon reasonable request.

## Supplementary Materials

The following are available online at https://www.mdpi.com/article/10.3390/cancers13225829/s1, Table S1: Frequency of symptoms after first and second dose in all patients, in those enrolled in early-phase trials, and in patients treated with immunotherapy, Table S2: Adverse events after the first and the second dose, according to the interval between vaccine administration and the administration of antineoplastic treatments, Table S3: Association between the incidence of adverse events after each vaccine dose and being enrolled in an early-phase trial, treatment with immunotherapy, and interval between vaccination and previous antineoplastic treatment. Results from univariate logistic regression models, Figure S1: Co-occurrence of adverse events after each vaccine dose in all patients, those enrolled in early-phase trials, and in patients treated with immunotherapy.

## References

[1] Barrière J., Gal J., Hoch B., Cassuto O., Leysalle A., Chamorey E., Borchiellini D. (2021). Acceptance of SARS-CoV-2 vaccination among French patients with cancer: A cross-sectional survey. Ann. Oncol..

[2] Polack F.P., Thomas S.J., Kitchin N., Absalon J., Gurtman A., Lockhart S., Perez J.L., Pérez M.G., Moreira E.D., Zerbini C. (2020). Safety and efficacy of the BNT162b2 mRNA Covid-19 vaccine. N. Engl. J. Med..

[3] Jackson L.A., Anderson E.J., Rouphael N.G., Roberts P.C., Makhene M., Coler R.N., McCullough M.P., Chappell J.D., Denison M.R., Stevens L.J. (2020). An mRNA Vaccine against SARS-CoV-2—Preliminary Report. N. Engl. J. Med..

[4] Anderson E.J., Rouphael N.G., Widge A.T., Jackson L.A., Roberts P.C., Makhene M. (2020). Safety and Immunogenicity of SARS-CoV-2 mRNA-1273 Vaccine in Older Adults. N. Engl. J. Med..

[5] Baden L.R., El Sahly H.M., Essink B., Kotloff K., Frey S., Novak R., Diemert D., Spector S.A., Rouphael N., Creech C.B. (2021). Efficacy and Safety of the mRNA-1273 SARS-CoV-2 Vaccine. N. Engl. J. Med..

[6] Voysey M., Clemens S.A.C., Madhi S.A., Weckx L.Y., Folegatti P.M., Aley P.K., Angus B., Baillie V.L., Barnabas S.L., Bhorat Q.E. (2020). Safety and efficacy of the ChAdOx1 nCoV-19 vaccine (AZD1222) against SARS-CoV-2: An interim analysis of four randomised controlled trials in Brazil, South Africa, and the UK. Lancet.

[7] Kuderer N.M., Choueiri T.K., Shah D.P., Shyr Y., Rubinstein S.M., Rivera D.R., Shete S., Hsu C.-Y., Desai A., Lopes G.D.L. (2020). Clinical impact of COVID-19 on patients with cancer (CCC19): A cohort study. Lancet.

[8] Desai A., Gupta R., Advani S., Ouellette L., Kuderer N.M., Lyman G.H., Li A. (2020). Mortality in hospitalized patients with cancer and coronavirus disease 2019: A systematic review and meta-analysis of cohort studies. Cancer.

[9] Tagliamento M., Agostinetto E., Bruzzone M., Ceppi M., Saini K.S., de Azambuja E., Punie K., Westphalen C.B., Morgan G., Pronzato P. (2021). Mortality in adult patients with solid or hematological malignancies and SARS-CoV-2 infection with a specific focus on lung and breast cancers: A systematic review and meta-analysis. Crit. Rev. Oncol..

[10] Corti C., Crimini E., Tarantino P., Pravettoni G., Eggermont A.M., Delaloge S., Curigliano G. (2021). SARS-CoV-2 vaccines for cancer patients: A call to action. Eur. J. Cancer.

[11] Sadoff J., Le Gars M., Shukarev G., Heerwegh D., Truyers C., de Groot A.M., Stoop J., Tete S., Van Damme W., Leroux-Roels I. (2021). Interim Results of a Phase 1–2a Trial of Ad26.COV2.S Covid-19 Vaccine. N. Engl. J. Med..

[12] Trapani D., Curigliano G. (2021). COVID-19 vaccines in patients with cancer. Lancet Oncol..

[13] Spiera R., Jinich S., Jannat-Khah D. (2021). Rituximab, but not other antirheumatic therapies, is associated with impaired serological response to SARS- CoV-2 vaccination in patients with rheumatic diseases. Ann. Rheum. Dis..

[14] Monin L., Laing A.G., Muñoz-Ruiz M., McKenzie D.R., del Molino del Barrio D., Alaguthurai T., Domingo-Vila C., Hayday T.S., Graham C., Seow J. (2021). Safety and immunogenicity of one versus two doses of the COVID-19 vaccine BNT162b2 for patients with cancer: Interim analysis of a prospective observational study. Lancet Oncol..

[15] Au L., Fendler A., Shepherd S.T.C., Rzeniewicz K., Cerrone M., Byrne F., Carlyle E., Edmonds K., Del Rosario L., Shon J. (2021). Cytokine release syndrome in a patient with colorectal cancer after vaccination with BNT162b2. Nat. Med..

[16] Garassino M.C., Vyas M., de Vries E., Kanesvaran R., Giuliani R., Peters S. (2021). The ESMO Call to Action on COVID-19 vaccinations and patients with cancer: Vaccinate. Monitor. Educate. Ann. Oncol..

[17] Desai A., Gainor J.F., Hegde A., Schram A.M., Curigliano G., Pal S., Liu S.V., Halmos B., Groisberg R., Grande E. (2021). Author Correction: COVID-19 vaccine guidance for patients with cancer participating in oncology clinical trials. Nat. Rev. Clin. Oncol..

[18] A Yap T., Siu L.L., Calvo E., Lolkema M.P., LoRusso P.M., Soria J.-C., Plummer R., de Bono J.S., Tabernero J., Banerji U. (2021). SARS-CoV-2 vaccination and phase 1 cancer clinical trials. Lancet Oncol..

[19] National Institutes of Health, National Cancer Institute (2017). Common Terminology Criteria for Adverse Events (CTCAE) Version 5.0.

[20] ASCO (2021). Inclusion of Individuals with Cancer on COVID-19 Vaccine Trials.

[21] Patel M.K., Bergeri I., Bresee J.S., Cowling B.J., Crowcroft N.S., Fahmy K., Hirve S., Kang G., Katz M.A., Lanata C.F. (2021). Evaluation of post-introduction COVID-19 vaccine effectiveness: Summary of interim guidance of the World Health Organization. Vaccine.

[22] WHO (2021). COVID-19 and Mandatory Vaccination: Ethical Considerations and Caveats. Policy Br..

[23] Centers for Disease Control and Prevention (CDC) Different COVID-19 Vaccines.

[24] Waissengrin B., Agbarya A., Safadi E., Padova H., Wolf I. (2021). Short-term safety of the BNT162b2 mRNA COVID-19 vaccine in patients with cancer treated with immune checkpoint inhibitors. Lancet Oncol..

[25] Mazzarella L., Duso B.A., Trapani D., Belli C., D’Amico P., Ferraro E., Viale G., Curigliano G. (2019). The evolving landscape of ‘next-generation’ immune checkpoint inhibitors: A review. Eur. J. Cancer.

[26] Hosseini I., Gadkar K., Stefanich E., Li C.-C., Sun L.L., Chu Y.-W., Ramanujan S. (2020). Mitigating the risk of cytokine release syndrome in a Phase I trial of CD20/CD3 bispecific antibody mosunetuzumab in NHL: Impact of translational system modeling. npj Syst. Biol. Appl..

[27] Mazzarella L., Giugliano S., D’Amico P., Belli C., Duso B.A., Rescigno M., Curigliano G. (2020). Evidence for interleukin 17 involvement in severe immune-related neuroendocrine toxicity. Eur. J. Cancer.

[28] Ceschi A., Noseda R., Palin K., Verhamme K. (2020). Immune Checkpoint Inhibitor-Related Cytokine Release Syndrome: Analysis of WHO Global Pharmacovigilance Database. Front. Pharmacol..

[29] Addeo A., Shah P.K., Bordry N., Hudson R.D., Albracht B., Di Marco M., Kaklamani V., Dietrich P.-Y., Taylor B.S., Simand P.-F. (2021). Immunogenicity of SARS-CoV-2 messenger RNA vaccines in patients with cancer. Cancer Cell.

