# Supplementary material for: Safety of COVID-19 mRNA Vaccines in Patients with Cancer Enrolled in Early-Phase Clinical Trials
Source: Cancers (Basel). 2021 Nov 20;13(22):5829. doi: 10.3390/cancers13225829 (PMC8616209; doi:10.3390/cancers13225829)
Supplement: Supplementary file 1 [file cancers-13-05829-s001.zip › cancers-1453383-supplementary.pdf]

Supplementary Materials: Safety of COVID-19 mRNA Vaccines in Patients with Cancer Enrolled in Early-Phase Clinical Trials

Pamela Trillo Aliaga, Dario Trapani, José Luis Sandoval, Edoardo Crimini, Gabriele Antonarelli, Grazia Vivanet, Stefania Morganti, Chiara Corti, Paolo Tarantino, Alex Friedlaender, Carmen Belli, Ida Minchella, Marzia Locatelli, Angela Esposito, Carmen Criscitiello and Giuseppe Curigliano

Table S1. Frequency of symptoms after first and second dose in all patients, in those enrolled in early phase trials and in patients treated with immunotherapy.

	**After 1st Vaccine Dose**			**After 2nd Vaccine Dose**		
Symptoms	**All (*n* = 113)**	**In early phase trials (*n* = 40)**	**IO (*n* = 20)**	**All (*n* = 113)**	**In early phase trials (*n* = 40)**	**IO (*n* = 20)**
Site pain	65.5% (*n* = 74)	65% (*n* = 26)	60% (*n* = 12)	58.4% (*n* = 66)	62.5% (*n* = 25)	60% (*n* = 12)
Fatigue	12.4% (*n* = 14)	10% (*n* = 4)	10% (*n* = 2)	22.1% (*n* = 25)	25% (*n* = 10)	20% (*n* = 4)
Fever	8.8% (*n* = 10)	7.5% (*n* = 3)	15% (*n* = 3)	18.6% (*n* = 21)	12.5% (*n* = 5)	20% (*n* = 4)
Headache	4.4% (*n* = 5)	5% (*n* = 2)	5% (*n* = 1)	7.1% (*n* = 8)	2.5% (*n* = 1)	5% (*n* = 1)
Myalgia	4.4% (*n* = 5)	0% (*n* = 0)	0% (*n* = 0)	8.8% (*n* = 10)	10% (*n* = 4)	5% (*n* = 1)
Other Aes	4.4% (*n* = 5)	5% (*n* = 2)	5% (*n* = 1)	2.7% (*n* = 3)	2.5% (*n* = 1)	5% (*n* = 1)
Site swelling	2.7% (*n* = 3)	2.5% (*n* = 1)	0% (*n* = 0)	4.4% (*n* = 5)	5% (*n* = 2)	0% (*n* = 0)
Arthralgia	2.7% (*n* = 3)	0% (*n* = 0)	0% (*n* = 0)	6.2% (*n* = 7)	5% (*n* = 2)	10% (*n* = 2)
Nausea	2.7% (*n* = 3)	0% (*n* = 0)	0% (*n* = 0)	6.2% (*n* = 7)	2.5% (*n* = 1)	5% (*n* = 1)
Site erythema	1.8% (*n* = 2)	0% (*n* = 0)	0% (*n* = 0)	2.7% (*n* = 3)	2.5% (*n* = 1)	0% (*n* = 0)
Shivers	0.9% (*n* = 1)	2.5% (*n* = 1)	5% (*n* = 1)	4.4% (*n* = 5)	5% (*n* = 2)	10% (*n* = 2)
Vomit	0.9% (*n* = 1)	0% (*n* = 0)	0% (*n* = 0)	0.9% (*n* = 1)	0% (*n* = 0)	0% (*n* = 0)
Site hardening	0% (*n* = 0)	0% (*n* = 0)	0% (*n* = 0)	1.8% (*n* = 2)	0% (*n* = 0)	0% (*n* = 0)
Lymp node swelling	0% (*n* = 0)	0% (*n* = 0)	0% (*n* = 0)	2.7% (*n* = 3)	2.5% (*n* = 1)	0% (*n* = 0)

Adverse events (AEs).

Table S2. Adverse events after the first and the second dose, according to the interval between the vaccine administration and the administration of antineoplastic treatments.

	**After 1st vaccine dose**			**After 2nd vaccine dose**		
Symptoms	**Same day (*n* = 29)**	**1–7days (*n* = 29)**	**>7days (*n* = 54)**	**Same day (*n* = 32)**	**1–7days (*n* = 24)**	**>7days (*n* = 56)**
Site pain	75.9% (*n* = 22)	51.7% (*n* = 15)	66.7% (*n* = 36)	53.1% (*n* = 17)	45.8% (*n* = 11)	66.1% (*n* = 37)
Fatigue	17.2% (*n* = 5)	6.9% (*n* = 2)	13% (*n* = 7)	28.1% (*n* = 9)	20.8% (*n* = 5)	19.6% (*n* = 11)
Myalgia	6.9% (*n* = 2)	6.9% (*n* = 2)	1.9% (*n* = 1)	15.6% (*n* = 5)	16.7% (*n* = 4)	1.8% (*n* = 1)
Site erythema	3.4% (*n* = 1)	0% (*n* = 0)	1.9% (*n* = 1)	0% (*n* = 0)	8.3% (*n* = 2)	1.8% (*n* = 1)
Fever	3.4% (*n* = 1)	10.3% (*n* = 3)	11.1% (*n* = 6)	15.6% (*n* = 5)	12.5% (*n* = 3)	23.2% (*n* = 13)
Headache	3.4% (*n* = 1)	6.9% (*n* = 2)	3.7% (*n* = 2)	6.3% (*n* = 2)	4.2% (*n* = 1)	8.9% (*n* = 5)
Arthralgia	3.4% (*n* = 1)	3.4% (*n* = 1)	1.9% (*n* = 1)	9.4% (*n* = 3)	8.3% (*n* = 2)	3.6% (*n* = 2)
Other AEs	3.4% (*n* = 1)	0% (*n* = 0)	7.4% (*n* = 4)	3.1% (*n* = 1)	4.2% (*n* = 1)	1.8% (*n* = 1)
Site swelling	0% (*n* = 0)	0% (*n* = 0)	5.6% (*n* = 3)	0% (*n* = 0)	12.5% (*n* = 3)	3.6% (*n* = 2)
Site hardening	0% (*n* = 0)	0% (*n* = 0)	0% (*n* = 0)	3.1% (*n* = 1)	4.2% (*n* = 1)	0% (*n* = 0)
Lymph node swelling	0% (*n* = 0)	0% (*n* = 0)	0% (*n* = 0)	0% (*n* = 0)	4.2% (*n* = 1)	3.6% (*n* = 2)
Shivers	0% (*n* = 0)	0% (*n* = 0)	1.9% (*n* = 1)	6.3% (*n* = 2)	12.5% (*n* = 3)	0% (*n* = 0)
Nausea	0% (*n* = 0)	6.9% (*n* = 2)	1.9% (*n* = 1)	6.3% (*n* = 2)	8.3% (*n* = 2)	5.4% (*n* = 3)
Vomit	0% (*n* = 0)	3.4% (*n* = 1)	0% (*n* = 0)	0% (*n* = 0)	0% (*n* = 0)	1.8% (*n* = 1)

Adverse events (AEs).

Table S3. Association between the incidence of adverse events after each vaccine dose and being enrolled in an early phase trial, treatment with immunotherapy and interval between vaccination and previous antineoplastic treatment. Results from univariate logistic regression models.

	**AEs**				**Local AEs**				**Systemic AEs**			
	**After 1st dose**		**After 2nd dose**		**After 1st dose**		**After 2nd dose**		**After 1st dose**		**After 2nd dose**	
	**OR [95%CI]**	***p***	**OR [95%CI]**	***p***	**OR [95%CI]**	***p***	**OR [95%CI]**	***p***	**OR [95%CI]**	***p***	**OR [95%CI]**	***p***
In early phase trials vs. not	1.1 [0.4;2.6]	0.9	1 [0.4;2.4]	1	1.2 [0.5;2.9]	0.65	1.2 [0.6;2.8]	0.62	1 [0.4;2.4]	0.92	0.6 [0.2;1.3]	0.19
No IO vs. IO	1.3 [0.4;3.7]	0.63	1.2 [0.4;3.3]	0.8	1.5 [0.5;4]	0.45	1.4 [0.5;3.8]	0.48	0.6 [0.2;2]	0.42	1.5 [0.6;4.7]	0.42
Vaccine >7 days after treatment vs. day of treatment	0.7 [0.2;2]	0.47	0.5 [0.1;1.5]	0.2	0.6 [0.2;1.6]	0.3	0.6 [0.2;1.4]	0.24	1.1 [0.4;3.3]	0.86	0.6 [0.3;1.6]	0.32
Vaccine 1-7 days after treatment vs. day of treatment	0.3 [0.1;1.1]	0.08	0.4 [0.1;1.3]	0.1	0.3 [0.1;0.9]	0.03	0.5 [0.2;1.4]	0.18	0.7 [0.2;2.4]	0.52	0.5 [0.2;1.4]	0.18

Adverse events (AEs), odds ratio (OR), confidence interval (CI), immunotherapy (IO).

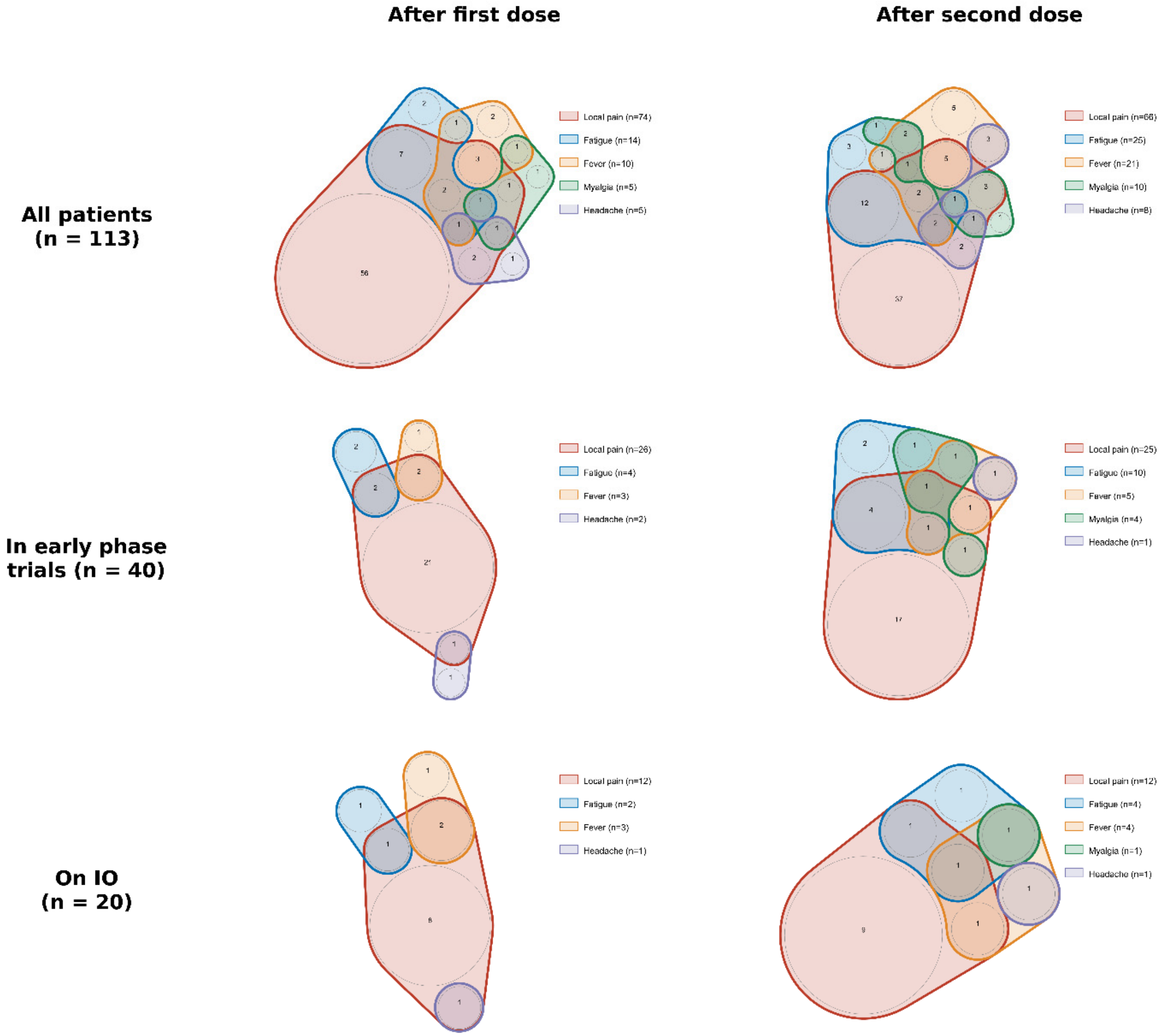

Figure S1. Co-occurrence of adverse events after each vaccine dose in all patients, those enrolled in early phase trials and in patients treated with immunotherapy.
